# Bridging Epidemiological Data with Features of the Urban Context: An experience of Urban Public Health within the City of Milan, Italy

**DOI:** 10.1093/eurpub/ckac129.705

**Published:** 2022-10-25

**Authors:** A Rebecchi, AA Johnson, A Brambilla, M Buffoli, AG Russo, S Capolongo

**Affiliations:** 1 Design & Health Lab, DABC - Politecnico di Milano, Milan, Italy; 2 Columbia University, Vagelos College of Physicians and Surgeons, New York, USA; 3 ATS della Città Metropolitana di Milano, Milan, Italy

## Abstract

Referring to the Research Project “Enhancing Healthcare and Well-Being Through the Potential of Big Data: An Integration of Survey, Administrative, and Open Data to Assess Health Risk in the City of Milan with Data Science” the Authors present preliminary results regarding a survey distributed to a sample of citizens across all neighborhoods of Milano city. This survey sought to collect data regarding health risk factors of this population, including both individual (e.g. socio-demographic characteristics, behaviors, etc.) and community (e.g. environmental/morphological features, available social services, etc.) data. A digital survey was designed to collect information on the health conditions, risk factors, and lifestyle characteristics of a representative sample of the Milanese population at the neighborhood level, with reference to the census tracts and Local Identity Units (NIL). Collected survey data are entered into a system containing corresponding individual health information acquired from the Local Health Authority databases, creating a synthesized information profile with each respondent's state of health, including existing conditions, health services used, and drug therapies. The disseminated survey was developed from comparisons with similar experiences at the national/international level and divided into 60 multiple choice questions (6 for Sociodemographic profile; 8 for Context of residence; 12 for Functional limitations; 25 for Behaviors and lifestyles; 9 for Access to health services). The data from urban analysis conducted on the NIL of the City of Milan are assessed with particular reference to the theme of bicycle-pedestrian accessibility (Walkability) in the urban context and repercussions on the adoption of Healthy Lifestyles. The models developed through this research are expected to provide critical insight for designing health promotion, health protection, and disease prevention interventions aimed both at individual and community level.

